# Dental Education. No. 2

**Published:** 1867-10

**Authors:** 


### ARTICLE III.

### *Dental Education. No.* 2.

By Professor Austen.

In the first paper of this series, it was shown, briefly—that education was something more than mere instruction: that it implied the co-operation of the student and demanded a measure of talent and industry in the taught, as well as in the teacher; and that professional education could not be effective without preparatory mental training. Hence the definition given. "The selection and preparation of professional material." The importance of selection, as preliminary to the education of students has been shown and in the next paper some of the methods of education will be considered. It is proposed at present to inquire into the means taken by a profession to discriminate between its members: also the object and wisdom of such discrimination.

It is scarcely consistent with American ideas of liberty to forbid any one the practice of any profession he may fancy himself equal to. In the absence of governmental restrictions, the professions must depend, for the maintenance of their standard, upon the carefulness with which they recognize the claims of membership. To this end societies, associations and colleges are established, laying down the conditions upon which, respectively, they are willing to acknowledge "fraternity and equality" and most of them issuing certificates of such acknowledgment—termed diplomas. Such diplomas have a value purely relative; and the letters, which they permit one to affix to his name, have no significance, apart from the body which grants the permission.

Associations for mutual improvement are valuable as educational instruments; but their diplomas have no meaning except as certificates of membership, unless they are based upon examination: or unless they are given in unsought acknowledgment of talent and position. Diplomas of this class measure the judgment of the grantors in the *selection*, not the *preparation* of professional material.

Such associations have doubtless a right to establish the conditions upon which they will pronounce men worthy of their honors. The worth of the honor is quite another question. One might be justly proud if elected "Corresponding Member" of the French Academy: yet not care to boast of an F. C. S. granted by some obseqious, patron-seeking, mushroom society. An L. L. D. should imply in its possessor a decent regard for, and a very thorough knowledge of, law: but what significance can these letters have when affixed by a sycophantic institution to some Bombastes Furioso, who notoriously tramples upon every law, human and Divine!

We have here *two* classes of diplomas. ONE, a simple certificate of membership, implying that its possessor is desirous to benefit himself by interchange of ideas; communicating, it may be hoped, as freely as he receives. Such diploma has a value, as distinguishing a man from that unprogressive class, which thinks it has nothing to learn and keeps what it knows to itself. It is evidence of one step taken in education and progress. As an evidence of professional ability it is absolutely worthless. The OTHER asserts for its owner the possession of superior qualification. This assertion rests for its value upon the consciencious truthfulness and professional knowledge and acumen of the examining body. Hence, such diplomas have values of extreme diversity, according to the sources whence they issue.

A THIRD class of diplomas is the award of some teacher or body of teachers. It certifies that the person named therein has creditably passed examination, after a prescribed course of study and practice, such as, in the judgment of the teacher or teachers, is sufficient to warrant admission into the professional ranks. The teacher assures the profession and the community that the student has for a specified time been engaged in study, and has given satisfactory evidence that he has made diligent use of his time. These diplomas have also values widely different, according to the estimation in which their grantors are held. They have a character wholly different from the two first, and should bear upon their face some mark of this distinction. In their influence upon professional advancement they are by far the most important, and the persons or associations conferring them should be careful how they mar their influence, or create confused ideas of their true character, by conferring degrees of any other kind.

Pursuing the analysis further, these three classes may be embraced under two divisions. 1st.—Complimentary acknowledgment of merit. 2nd.—Certificate of the faithful pursuance of a certain course of professional study.

The first class may, in the comparatively new profession of Dentistry, be considered as a well earned tribute to its pioneers. Also, if charily and judiciously conferred upon acknowledged high skill and ability, or for distinguished service, it may be useful as an incentive to active genius in the rising generation. It should be so wholly different from the second class as by no possibility to be confounded with it: but rather become an object of ambition to those who hold the latter. If it convey a title, or add initials, these should be distinctive and the parchment itself should present a widely different appearance.

It may be very philosophical to despise titles and speak contemptiously of "bits of colored ribbon." The fact remains that appreciation of worth is very sweet to the wisest and greatest, and its tokens are highly esteemed. It is only when we seek the title or ribbons without possessing the worth, that we become justly the butt of the philosophers. And when conferred indiscriminately or profusely they are emphatically "empty letters and bits of silk," not worth the acceptance of any sensible man. Specially contemptible are such honors, when confered by any literary or professional institution, with a view to secure influence, or lift itself into notoriety.

Honorary Diplomas, medals or ribbons are worthy objects of ambition in proportion to their value, which depends solely upon the grantor. Knighthood is most prized when coming from that monarch, who is chary in giving, with his sword, the stroke of honor. Social distinctions, marked by garters and ribbons, are always prized, when coming from "majesty," the fountain of social honor. But when a professional man exhibits his array of gold snuff-boxes, diamond rings and medals, the gifts of crowned heads, in evidence of his professional skill, we say, with the old painter "*ne sutor supra crepidem.*" Yet Victoria's grateful memento for the relief of a tooth-ache is worth even more, as a token of merit, than the Diplomas of Institutions, which confer the same to gain prestige or popularity.

There is a third kind of diploma conferred by some colleges upon "examination" of Dentists, who have been in practice for a certain number of years. It is neither the award of distinguished merit nor the certificate of the well drilled tyro. It gives none of the eclat of an honorary degree, and it robs the student's degree of its meaning, by being confounded therewith. Its occasional occurrence, under peculiar circumstances may be perhaps excused or palliated. Its adoption, as a principle, by any Institution, should be deplored, as tending to foster in the profession an unworthy craving after title for title sake.

If this degree is judicious the plan cannot be too universally carried out. Now let every Medical school advertize for candidates, who are willing, without pursuing a specified curriculum of study, to undergo the ordeal of examination, as severe as the average of such trials by medical Faculties—the only requisite being that they shall have practiced medicine for a certain term of years—how, or where, or upon what principle it matters not, if only the momentous questions crowded into a few hours examination are satisfactorily answered. There is not a high medical authority in the country, but would pronounce this step a retrograde movement, most injurious to the cause of medical education. That there are so many inferior medical schools; that the majority of medical graduates should tremble before a board of Army and Navy Examiners; that we trust the lives of our wives and children to practitioners, who would not be considered competent to attend soldiers and sailors: that most Prussian graduates, just out of the schools, have a better knowledge of the theory and practice of medicine than many of our medical Professors is all bad enough, without converting a host of charlatans into Doctors of Medicine by the magic wand of a brief examination.

If bad in Medicine, the scheme is bad also in Dentistry; although the position of the two, relative to titles and colleges is different. The untitled physician ranks with charlatans; for within the memory of this generation, graduation in some school has been universally regarded as an essential pre-requisite to honorable practice. But the oldest (Baltimore) Dental College dates back only to 1840. The physician who can show no diploma looses caste, because of the inference (wrong only in a few exceptional cases) that he has not used all available means of acquiring a knowledge of his profession. But no dentist 30 years ago had any dental title; comparatively few had a medical degree. The profession was imperfectly organized and its terms of initiation vague. This class of dentists hold their reputation independent of any dental diplomas. Some among them may have accepted Honorary degrees; but the remainder lose no caste by the absence of a kind of degree, which, if universal, is no longer honorary. These SENIOR members of the profession compromise the dignity of their position, when they seek the honors granted by Dental Colleges.

The same remarks apply with nearly equal force to all dentists in practice during the ten years (1840—1850) when collegiate training was contending for an acknowledgment of its necessity. Of this second class, many, after years of practice, attended lectures in dental colleges. Their titles are an honorable evidence of their earnestness in the pursuit of professional knowledge: yet no occasion of reproach to those who did not feel this necessity. Whilst the first class are above the benefit of the diploma, the second class may rightly hold themselves indifferent to it.

Of a third class—those entering the profession, (1850—60) when the number of colleges had increased, and their importance very generally acknowleged—it may still be said, absence of title is no disgrace. But from this point let the lines be drawn: for the purpose of fairly testing most important questions of dental education, and not in any spirit of rivalry or disparagement.

Let the D.D.S. mark the practitioner, of whose training one or two sessions in dental colleges has formed an essential part. Let the M.D. mark those who have studied dentistry as a medical specialty; and let the absence of title mark those, who are content with the experience gained in an offiee. Let each preserve its distinct insignia and neither seek to borrow the badge of the others.

When the profession and community shall have decided that any one of these systems, say the collegiate, is the best and should form an essential part of dental education, then may the medical law be applied and all untitled practitioners be ostracised.

Meanwhile let dental colleges refuse the degree to all who do not actually receive instruction within their walls. Let them be restricted to their specific duty—the education of students ; doing this in the best possible manner, using their diploma simply and solely as an evidence of that fact ; and leaving to the future all decisions as to the superiority of their teaching. Used in this way, the diploma has a very important significance, assuring a more speedy solution of the educational problem.

When a college quits this, its proper sphere of action and sits as judge upon dental practitioners, not its students, it arouses miserable jealousies, robs its own diploma of its true import and takes from the regular student a laudable incentive. It may thus widen its circle of active patrons and increase its list of students and graduates. But whilst apparently making the collegiate system more popular, it brings it into disfavour with a large class of best practitioners, who deem it an insufferable arrogance in any collegiate faculty to cast this implied slur upon their professional position. For as matter of course, if the seal of this examining body gives *eclat*, its absence is a reproach. Differing essentialy in this respect from the student's diploma and the honorary degree, it becomes a mischievous source of ill feeling, calculated to bring all diplomas into disrepute.

A society may with propriety issue its certificates of membership. An association may adopt forms of examination to prevent the intrusion of unworthy members. A college may issue its certificates of faithful attendance upon prescribed courses of study. They may all occasionally compliment talent and skill by parchment or medal. These are legitimate exercises of privilege ; but when any one of them calls upon the profession at large to present themselves "for examination," it provokes inquiry into the peculiar attributes of this professional Supreme Court, by whose decisions they are to stand or fall. If a congress of dentists should meet and appoint a committee of examiners, they might award a diploma which would serve as a sort of "standard measure:" but the attempt of any self-chosen board of review to apply such a standard will be resisted. It is an injustice to the cause of education to connect it in any way with such unwarrantable assumption of superiority. Earnestly protesting against such a combination, as being opposed to the true aim of dental education, we pass to the consideration of some of its methods and instruments.

(To be Continued.)

---

### ARTICLE IV.

### *Dentistry as a Fine Art. No.* 5.

